# Phase I Study Assessing the Pharmacokinetic Profile, Safety, and Tolerability of a Single Dose of Ceftazidime-Avibactam in Hospitalized Pediatric Patients

**DOI:** 10.1128/AAC.00862-16

**Published:** 2016-09-23

**Authors:** John S. Bradley, Jon Armstrong, Antonio Arrieta, Raafat Bishai, Shampa Das, Shirley Delair, Timi Edeki, William C. Holmes, Jianguo Li, Kathryn S. Moffett, Deepa Mukundan, Norma Perez, José R. Romero, David Speicher, Janice E. Sullivan, Diansong Zhou

**Affiliations:** a University of California, San Diego, California, USA; b AstraZeneca, Macclesfield, United Kingdom; c Children's Hospital of Orange County, Orange, California, USA; d AstraZeneca, Gaithersburg, Maryland, USA; e Children's Hospital & Medical Center, Omaha, Nebraska, USA; f AstraZeneca, Wilmington, Delaware, USA; g AstraZeneca, Waltham, Massachusetts, USA; h West Virginia University, Morgantown, West Virginia, USA; i University of Toledo, Toledo, Ohio, USA; j University of Texas Health Science Center, Houston, Texas, USA; k University of Arkansas for Medical Sciences and Arkansas Children's Hospital, Little Rock, Arkansas, USA; l University Hospitals Rainbow Babies and Children's Hospital, Cleveland, Ohio, USA; m University of Louisville and Kosair Children's Hospital, Louisville, Kentucky, USA

## Abstract

This study aimed to investigate the pharmacokinetics (PK), safety, and tolerability of a single dose of ceftazidime-avibactam in pediatric patients. A phase I, multicenter, open-label PK study was conducted in pediatric patients hospitalized with an infection and receiving systemic antibiotic therapy. Patients were enrolled into four age cohorts (cohort 1, ≥12 to <18 years; cohort 2, ≥6 to <12 years; cohort 3, ≥2 to <6 years; cohort 4, ≥3 months to <2 years). Patients received a single 2-h intravenous infusion of ceftazidime-avibactam (cohort 1, 2,000 to 500 mg; cohort 2, 2,000 to 500 mg [≥40 kg] or 50 to 12.5 mg/kg [<40 kg]; cohorts 3 and 4, 50 to 12.5 mg/kg). Blood samples were collected to describe individual PK characteristics for ceftazidime and avibactam. Population PK modeling was used to describe characteristics of ceftazidime and avibactam PK across all age groups. Safety and tolerability were assessed. Thirty-two patients received study drug. Mean plasma concentration-time curves, geometric mean maximum concentration (*C*_max_), and area under the concentration-time curve from time zero to infinity (AUC_0–∞_) were similar across all cohorts for both drugs. Six patients (18.8%) reported an adverse event, all mild or moderate in intensity. No deaths or serious adverse events occurred. The single-dose PK of ceftazidime and avibactam were comparable between each of the 4 age cohorts investigated and were broadly similar to those previously observed in adults. No new safety concerns were identified. (This study has been registered at ClinicalTrials.gov under registration no. NCT01893346.)

## INTRODUCTION

In recent years there has been a global increase in the prevalence of multidrug-resistant Gram-negative pathogens in adults and children, including extended-spectrum β-lactamase and carbapenemase-producing Enterobacteriaceae. Longstanding carbapenem resistance has been documented in Pseudomonas aeruginosa since the approval and use of the first carbapenem, imipenem-cilastatin (1–5). There have also been reports of an increasing trend toward antibiotic resistance among Enterobacteriaceae isolated from children (6, 7). As a consequence of increasing resistance, the utility of many antibiotic classes (including the cephalosporins and carbapenems) to treat serious Gram-negative infections has become compromised (8–12), creating an urgent need for new antibiotic therapies that utilize novel mechanisms of action. In particular, very few options have been investigated for the treatment of complicated Gram-negative infections in pediatric patients, and although several novel agents are in clinical development, pharmacokinetic (PK) data in children and adolescents is limited at present (13).

The PK profile of drugs differs between pediatric and adult patients (14). Body size and physiology develop rapidly in the first few years of life, resulting in significant differences in the absorption, distribution, metabolism, and excretion of drugs between pediatric and adult patients (15). Studies are required to assess the PK, safety, and tolerability of new drugs in all pediatric age groups.

Ceftazidime is an extended-spectrum antipseudomonal cephalosporin that was first approved in 1985 for the treatment of complicated infections in adults, with approval for children shortly thereafter (16). Widespread β-lactamase-mediated resistance has greatly reduced cephalosporin effectiveness (17); therefore, the combination of ceftazidime with avibactam, a novel non-β-lactam β-lactamase inhibitor, represents an important new option for the treatment of serious Gram-negative infections (18–21). Avibactam has been shown *in vitro* to inactivate Ambler class A and C (including Klebsiella pneumoniae carbapenemases) and some class D β-lactamases, which restores the bactericidal activity of ceftazidime against otherwise ceftazidime-resistant pathogens carrying these β-lactamases. However, avibactam does not inhibit metallo-β-lactamases (22).

Ceftazidime-avibactam (2,000 mg and 500 mg, respectively) is approved for adults in the U.S. for the treatment of complicated intra-abdominal infections (cIAI), when used in combination with metronidazole, and complicated urinary tract infections (cUTI) with limited or no alternative treatment options (23). The efficacy and tolerability of ceftazidime-avibactam in the treatment of adults with cIAI and cUTI has been demonstrated in three recently reported phase III studies (NCT01499290, NCT01500239, and NCT01644643), none of which raised any new safety concerns (24, 25).

The primary objective of the phase I study reported here was to characterize the PK profile of a single dose of ceftazidime-avibactam in hospitalized infants and children with the aim of providing data to support future clinical studies of ceftazidime-avibactam in pediatric patients. Safety and tolerability were evaluated as secondary objectives.

(These data were presented in part at the 55th Interscience Conference on Antimicrobial Agents and Chemotherapy, 17 to 21 September 2015, San Diego, California [26].)

## MATERIALS AND METHODS

### Study design.

This was a phase I, multicenter, open-label, single-dose study (sponsor protocol number D4280C00014; ClinicalTrials.gov identifier NCT01893346) conducted in pediatric patients who were hospitalized with infections in the U.S. between July 2013 and October 2014. This was not a therapeutic study, and ceftazidime-avibactam was not used to treat the infection for which the patient had been hospitalized. The study was approved by the local Institutional Review Board of each of the investigators and was performed in accordance with the ethical principles that have their origin in the Declaration of Helsinki. The study is consistent with International Conference on Harmonization (ICH)/Good Clinical Practice (GCP) and applicable regulatory requirements and the AstraZeneca policy on bioethics.

### Patients.

Patients eligible for inclusion were hospitalized male or female children aged ≥3 months to <18 years who were receiving systemic antibiotic therapy for the treatment of a suspected or confirmed infection and who were expected to require hospitalization for at least 24 to 48 h. Hospitalization was mandatory for the first 24 h after infusion of ceftazidime-avibactam, with early discharge possible if the patient was able to return to the hospital/clinic for assessments on day 3. Patients were required to have sufficient intravenous (i.v.) access (peripheral or central) to receive the study drug and adequate access for PK sampling and to be likely to survive the current illness for which they were hospitalized. Prescribed antibiotics used to treat the patient's infection were to be continued without alteration.

Female subjects who had reached reproductive maturity were required to have a negative serum β-human chorionic gonadotropin test just prior to study entry and agree not to attempt pregnancy from the time of screening until 7 days after receipt of study drug. Key exclusion criteria were a history of hypersensitivity reactions to carbapenems, cephalosporins, penicillin, other β-lactam antibiotics, or metronidazole; a past or current history of epilepsy or seizure disorder (excluding childhood febrile seizures); severe renal impairment (creatinine clearance [CrCL] of ≤30 ml/min/1.73 m^2^); pregnancy or breastfeeding; acute hepatitis in the prior 6 months; a history of cirrhosis, acute hepatic failure, or acute decompensation of chronic hepatic failure; and any condition, such as septic shock, burns, or cystic fibrosis, that would make patients unsuitable for analysis of PK as the basis for dosing in proposed phase II treatment trials for cIAI and cUTI in children. Those with body mass index (BMI) below the 5th and above the 95th percentile based on age, height, and weight (excluding children <2 years old) were also excluded. Any patient who received ceftazidime within 12 h of study drug administration (24 h if CrCL was ≤50 ml/min) was also excluded. Patients (if age appropriate) provided assent to participate, and the parent or legal guardian for each patient provided written informed consent prior to any study-specific procedures.

### Treatment.

The study included 4 cohorts grouped according to regulatory guidance (FDA pediatric guidance 2014), and each comprised at least 8 evaluable patients: cohort 1, ≥12 to <18 years; cohort 2, ≥6 to <12 years; cohort 3, ≥2 to <6 years; and cohort 4, ≥3 months to <2 years. Cohort 4 excluded young infants born prior to 37 weeks of gestation.

Dose regimens used in the present study are summarized in Table 1. All doses were administered as a 2-h continuous i.v. infusion to maximize the pharmacodynamic (PD) exposure for β-lactam antibiotics (27), i.e., the time during which non-protein-bound (or “free”) antibiotic is present at the site of infection at concentrations that are above the MIC of the pathogen (*f*T>MIC) (28). Dosing in cohorts 1 and 2 was based on the probability of target attainment (PTA) of at least 90% by achieving the appropriate *f*T>MIC exposure in children in each age group, using population PK models of ceftazidime and avibactam that were developed from phase I and phase II studies in adults (29) and incorporating pediatric-specific changes in ceftazidime and avibactam PK according to allometric scaling for body weight (29). Dosing in cohorts 3 and 4 was based on updated population PK models of ceftazidime and avibactam using data from the older pediatric age groups in cohorts 1 and 2 and taking into account literature-reported maturation of renal function in children <2 years (30). The PK/PD targets used to determine doses for this study were based on those established in adults: ceftazidime free plasma concentrations above the ceftazidime-avibactam MIC (*f*T>MIC) of 8 mg/liter and avibactam free plasma concentrations above the critical threshold concentration (*f*T>*C_T_*) of 1 mg/liter, both for approximately 50% of the dosing interval (27–29). The PK/PD target of 50% *f*T>MIC is a well-characterized target for cephalosporins (31, 32), and a MIC of 8 mg/liter was selected for the ceftazidime target as it encompasses the MIC_90_s for Enterobacteriaceae (33, 34) and P. aeruginosa (21, 33, 35–37). The avibactam target was based on *f*T>*C_T_*, as this has previously been shown to be the PD index that most accurately predicts avibactam efficacy *in vitro* and *in vivo* (38, 39). A conservative *C_T_* of 1 mg/ml was selected for the PK/PD target to ensure adequate levels of β-lactamase inhibition to restore ceftazidime activity against representative ceftazidime-resistant strains of Enterobacteriaceae and P. aeruginosa (38, 39). The time above *C_T_* of 50% for avibactam was derived from preclinical studies (38) and also matches the ceftazidime target of 50% *f*T>MIC, thus protecting ceftazidime activity during the period it is most active. Doses were selected to achieve a PTA of at least 90%, with ceftazidime and avibactam exposures comparable with those in adults receiving ceftazidime-avibactam at 2,000 mg/500 mg every 8 h (q8h; the FDA-approved dose for adults based on phase III clinical trials), although not exceeding 20% higher than the simulated exposure (area under the concentration-time curve [AUC] and maximum plasma concentration [*C*_max_]) observed in adults (40, 41). Doses for cohorts 1 and 2 were determined from adult data; doses were subsequently determined for cohorts 3 and 4 based on data from cohorts 1 and 2. The final analysis, based on data from all available cohorts, supported the appropriateness of dose selection for each cohort during study implementation, with predicted PTA for the PK/PD target of 98% in cohort 1 and 94% to 98% for cohorts 2 to 4. Central clearance estimates for ceftazidime and avibactam in pediatric patients were estimated to be 26% and 30% greater, respectively, than for healthy adult volunteers. The PTAs for pediatric patients were predicted to be higher than the projected PTA in adult patients with normal renal function (90%), and the predicted exposure in *C*_max_ and AUC in pediatric subjects overall were within 30% of adult cIAI patients with mild renal impairment (42). Recruitment to cohorts 1 and 2 was conducted in parallel. All available data from cohorts 1 and 2 were reviewed by the safety review committee prior to proceeding with cohort 3. All available safety, tolerability, and predicted exposure data from cohort 3 were reviewed when making dosing decisions for cohort 4. Predicted exposures in cohort 3 and cohort 4 were also considered in all dosing decisions. Patients were monitored for 48 h following the end of study drug infusion.

**TABLE 1:** Summary of dose regimen used in the study

Drug	Dose (all administered as a 2-h i.v. infusion) for cohort:				
	1 (≥12 yr to <18 yr)	2 (≥6 yr to <12 yr)		3 (≥2 yr to <6 yr)	4 (≥3 mo to <2 yr)
		≥40 kg	<40 kg		
Ceftazidime	2,000 mg	2,000 mg	50 mg/kg	50 mg/kg	50 mg/kg
Avibactam	500 mg	500 mg	12.5 mg/kg	12.5 mg/kg	12.5 mg/kg

### Assessments. (i) Pharmacokinetics.

Blood samples (1 ml in cohorts 1 and 2, 0.5 ml in cohorts 3 and 4) were obtained for PK analysis at defined sampling times (end of infusion [EOI] ± 5 min and 30 min, 1.5 h, 3 h, 6 h, 10 h, and 22 h after EOI) for cohort 1 and specific sampling time windows for cohorts 2 to 4 (cohort 2, EOI ± 5 min and 15 to 45 min, 1 to 2 h, 2 to 3 h, 4 to 6 h, and 11 to 13 h after EOI; cohorts 3 and 4, EOI ± 5 min and 15 to 45 min, 2 to 3 h, and 4 to 6 h after EOI). A sparse sampling approach was used in cohorts 3 and 4 to minimize blood sampling requirements in these smaller patients.

Ceftazidime and avibactam plasma concentrations were determined by Covance Laboratories Limited (Harrogate, United Kingdom) using liquid chromatography with tandem mass spectrometry (LC-MS/MS) according to a previously reported validated methodology following solid-phase extraction (avibactam) or protein precipitation (ceftazidime) (43). All samples were analyzed within the known stability periods for ceftazidime and avibactam. The lower limit of quantification was 50 ng/ml for ceftazidime and 10 ng/ml for avibactam.

Ceftazidime and avibactam concentration-time courses were used to derive individual noncompartmental PK parameters for cohorts 1 and 2, including *C*_max_, AUC from time zero to last observed concentration (AUC_0–*t*_), AUC from time zero to infinity (AUC_0–∞_), elimination half-life (*t*_1/2_), volume of distribution at steady state (*V*_ss_), and clearance (CL). Due to sparse sampling in cohorts 3 and 4, ceftazidime and avibactam noncompartmental PK parameters could not be derived without population PK analysis. In these cohorts, plasma concentrations at the EOI were used as a comparable measurement for *C*_max_ in cohorts 1 and 2. Plasma concentrations were plotted based on the midpoint of the sampling time window for cohorts 2 to 4.

### (ii) Population pharmacokinetic modeling.

Using data from appropriate previous clinical studies in adults, a population PK model has been developed to describe ceftazidime and avibactam exposure (29). Ceftazidime and avibactam plasma concentrations, pediatric patient demographics, and disease status data from cohorts 1 to 4 of the present study were used to update this model (29, 42). From this updated model, individual PK profiles for pediatric patients with available ceftazidime and avibactam plasma concentration data were estimated using the empirical Bayesian *post hoc* estimates. Ceftazidime and avibactam AUC_0–∞_ values for cohorts 3 and 4 were derived from these population PK modeled profiles.

### (iii) Safety.

Safety and tolerability data were collected for each patient from the time of informed consent from the parent(s) or other legally acceptable representative(s) and informed assent from the patient, as appropriate, from day −1 or day 1 through to the day 3 follow-up visit. Safety and tolerability data were analyzed from time of first dose onward and were based on adverse event (AE) and serious AE reports, vital sign measurements (at baseline, at 2, 4, 6, and 12 h, and at 2 and 3 days after the EOI), electrocardiograms (at baseline and 15 min after EOI), physical examinations (at baseline and day 3), and clinical laboratory tests (at baseline and day 3). The clinical significance of laboratory results was determined by the investigator according to a predefined set of criteria. AEs were listed and tabulated by system organ class (SOC) and preferred term (PT) according to the Medical Dictionary for Regulatory Activities (MedDRA), version 16.1.

### Statistical analysis.

The planned sample size was based on typical PK characterization studies. No statistical calculations were performed to determine sample size. A minimum target of 32 patients was set for all cohorts collectively with at least 8 patients in each cohort. The PK analysis population included all patients who had received an i.v. study dose of ceftazidime-avibactam and had at least 1 postdose blood sample. An evaluable patient was defined as a patient who provided PK blood samples at ≥50% of the sampling time points. PK parameters were summarized by analyte and measurement time using descriptive statistics. All analyses were performed using Statistical Analysis Software (SAS, release 9.1 or higher) (SAS Institute, Inc., Cary, NC) or Phoenix WinNonlin version 6.2 or higher (Pharsight Corporation, MO). Population PK modeling was performed using the NONMEM software (version 7.2; ICON Development Solutions, Hanover, MD) running under PsN (Perl-speaks-NONMEM) 3.7.6 on a grid of CentOS 5.6 Linux servers, using the Intel Fortran compiler version 12.0.4. The safety analysis population included all patients who had received any amount of an i.v. study dose of ceftazidime-avibactam. No statistical tests were performed for any safety analyses.

## RESULTS

### Patients.

A total of 35 patients were enrolled across the 4 cohorts at 11 centers in the U.S. Three patients who were enrolled into cohort 1 did not receive any study medication (2 withdrew consent and 1 was withdrawn due to failing eligibility criteria). No patients were withdrawn due to AEs. Thirty-two patients (8 in each cohort) received the full ceftazidime-avibactam dose and 31 patients completed the study. Study completion was generally balanced across the 4 cohorts. One patient in cohort 2 did not attend the day 3 visit, and no follow-up data are available for this patient. Both the PK population and safety population comprised a total of 32 patients. Patient baseline demographic and clinical characteristics are summarized in Table 2. Excluding age, BMI, and CrCL, all patient baseline characteristics were similar across the 4 cohorts. Patients were predominantly white (*n* = 24), and all had normal renal function at baseline. There was a higher proportion of female patients in cohorts 1 and 3 and a higher proportion of male patients in cohorts 2 and 4.

**TABLE 2:** Baseline patient characteristics (safety analysis population)

Parameter	Value for cohort:			
	1 (≥12 to <18 yr) (*n* = 8)	2 (≥6 to <12 yr) (*n* = 8)	3 (≥2 to <6 yr) (*n* = 8)	4 (≥3 mo to <2 yr) (*n* = 8)
Age, yr				
Mean (SD)	14.9 (1.6)	8.0 (1.4)	3.5 (1.0)	0.9 (0.5)
Median (range)	14.9 (13.0–17.3)	7.7 (6.5–10.8)	3.8 (2.1–4.9)	0.9 (0.3–1.8)
Female, *n* (%)	5 (62.5)	3 (37.5)	6 (75.0)	3 (37.5)
Race, *n* (%)				
White	6 (75.0)	6 (75.0)	7 (87.5)	5 (62.5)
Black or African American	1 (12.5)	2 (25.0)	0	3 (37.5)
Other^*a*^	1 (12.5)	0	1 (12.5)	0
Weight, kg; mean (SD)	51.90 (7.15)	24.95 (3.03)	15.73 (2.39)	9.20 (2.51)
BMI, kg/m^2^; mean (SD)	20.80 (3.21)	15.41 (0.78)	15.47 (0.95)	NA^*d*^
CrCL, ml/min/1.73 m^2^; mean (SD)	115.29 (18.14)	156.97^*b*^ (42.92)	181.57 (100.99)	104.35 (26.20)
Normal renal function, *n* (%)	8 (100)	8 (100)	8 (100)	8 (100)
Any concomitant antibiotic,^*c*^ *n* (%)	8 (100)	8 (100)	8 (100)	8 (100)
Lincosamides	3 (37.5)	2 (25.0)	4 (50.0)	6 (75.0)
3rd-generation cephalosporins other than ceftazidime	1 (12.5)	3 (37.5)	1 (12.5)	1 (12.5)
Penicillins plus β-lactamase inhibitors	1 (12.5)	0	2 (25.0)	2 (25.0)
Vancomycin	1 (12.5)	1 (12.5)	0	2 (25.0)
Metronidazole	2 (25.0)	2 (25.0)	0	0
Aminoglycosides	2 (25.0)	1 (12.5)	0	1 (12.5)
1st-generation cephalosporins	1 (12.5)	0	1 (12.5)	1 (12.5)
Azithromycin	1 (12.5)	1 (12.5)	1 (12.5)	0
Extended-spectrum penicillin	1 (12.5)	1 (12.5)	1 (12.5)	0
Meropenem	0	2 (25.0)	0	0
Levofloxacin	0	0	2 (25.0)	0

a Asian (*n* = 1 in cohort 3 [≥2 to <6 years]) and American Indian or Alaskan native (*n* = 1 in cohort 1 [≥12 to <18 years]).

b One patient in cohort 2 (≥6 to <12 years) had a value greater than the upper limit of normal and is not included in the mean (SD) calculation.

c Taken any time between the initiation of study therapy and the day 3 follow-up assessment. Most commonly used antibiotics/groups are shown.

d NA, not applicable.

The most frequent current infections were cellulitis (15.6%), limb abscess (9.4%), and appendicitis (9.4%). All patients had received at least 1 prior antibiotic medication (data not shown) and received at least 1 concomitant antibiotic medication (Table 2). Overall, the most common antibiotic medications received were clindamycin (lincosamide) and third-generation cephalosporins (prior, 34.4% for both; concomitant, 46.9% and 18.8%, respectively). Of the 32 children enrolled in the study, 4 received concurrent vancomycin and 4 received an aminoglycoside.

### Pharmacokinetics.

**FIG 1:**
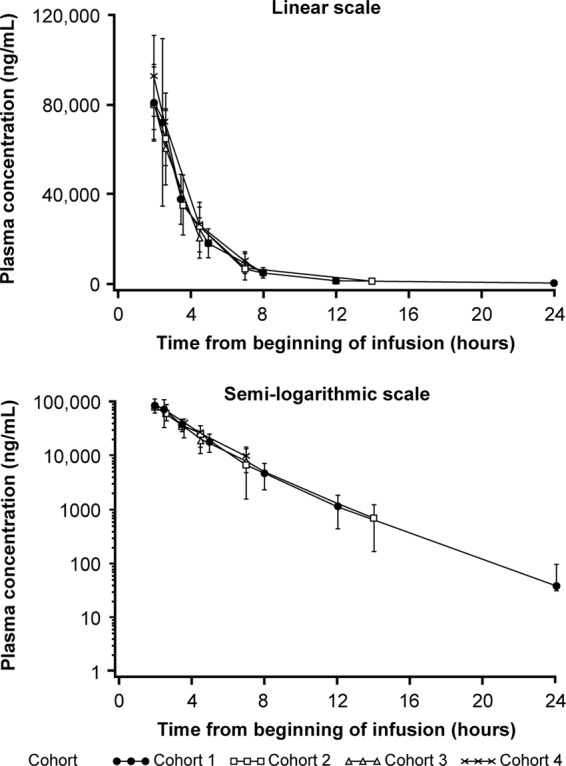
Arithmetic mean (±SD) plasma concentration-time curves for ceftazidime for cohorts 1 to 4 following single-dose (day 1) administration of ceftazidime-avibactam (pharmacokinetic analysis population).

**FIG 2:**
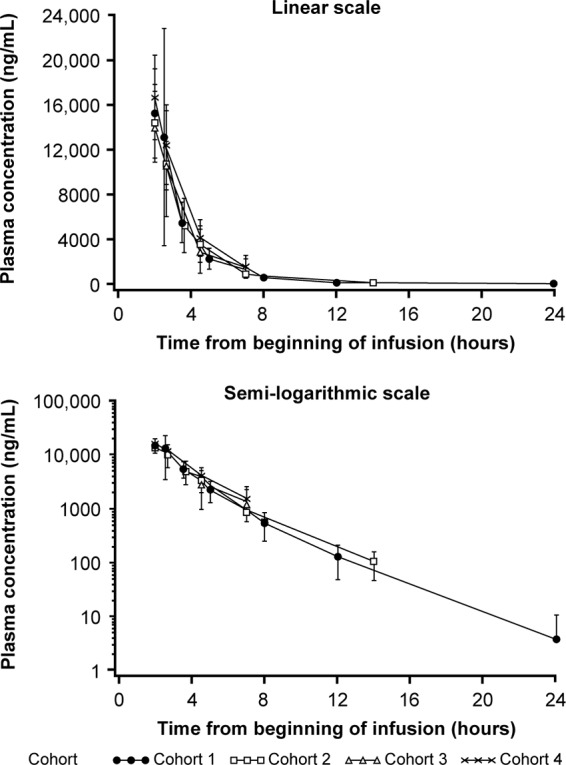
Arithmetic mean (±SD) plasma concentration-time curves for avibactam for cohorts 1 to 4 following single-dose (day 1) administration of ceftazidime-avibactam (pharmacokinetic analysis population).

Mean (SD) plasma concentration-time curves from the available data for ceftazidime and avibactam following single-dose administrations on day 1 were similar across cohorts 1 to 4 for both drugs (Fig. 1 and 2); due to sparse sampling of cohorts 3 and 4, the midpoints were used to plot the data to enable comparison between all cohorts. Geometric mean (percent coefficient of variation) *C*_max_ was similar across all four age cohorts for ceftazidime and avibactam (Table 3). Due to the sparse sampling, *C*_max_ was the only parameter measured for cohorts 3 (≥2 to <6 years) and 4 (≥3 months to <2 years). Median (range) time to maximum plasma concentration (*t*_max_) for cohorts 1 and 2 was similar and occurred immediately after EOI. The geometric mean AUC_0–*t*_ and AUC_0–∞_ were similar between cohorts 1 and 2 for both ceftazidime and avibactam. For both cohorts, the geometric mean value of AUC_0–*t*_ was approximately 99% of the AUC_0–∞_. Median (range) terminal *t*_1/2_ values for ceftazidime were similar between cohorts 1 and 2, 1.7 h (0.9 to 2.8) and 1.6 h (0.9 to 1.8), respectively. The geometric mean weight-normalized CL was slightly higher in cohort 2 than in cohort 1 (CL, 0.226 versus 0.169 liter/kg/h), while *V*_ss_ was lower in cohort 2 than cohort 1 (*V*_ss_, 13.0 versus 22.2 liters), although there was large within-cohort variability and CL was considered broadly comparable between cohorts (Table 3). Similar findings were also observed in cohort 1 and cohort 2 for median avibactam *t*_1/2_ (range) values of 1.6 h (0.9 to 2.8) and 1.7 h (0.9 to 2.0), respectively, and geometric mean CL and *V*_ss_ values (*t*_1/2_, 1.6 versus 1.7 h; CL, 13.7 versus 8.9 liters/h; *V*_ss_, 31.0 versus 19.3 liters). Like ceftazidime, geometric mean weight-normalized CL for avibactam differed between cohorts 1 and 2, with large within-cohort variability, but was considered broadly similar between cohorts (Table 3).

**TABLE 3:** Summary of ceftazidime and avibactam pharmacokinetic parameters measured in pediatric patients (pharmacokinetic population)

Drug and parameter^*a*^	Value for cohort:			
	1 (*n* = 8) (≥12 to <18 yr)	2 (*n* = 8) (≥6 to <12 yr)	3 (*n* = 8) (≥2 to <6 yr)	4 (*n* = 8) (≥3 mo to <2 yr)
Ceftazidime				
*C*_max_ (mg/liter)	79.8 (41.8)	81.3 (17.8)	80.1^*b*^ (14.7)	91.7^*b*^ (19.6)
*t*_max_^*c*^ (h)	2.0 (1.9–2.6)	2.1 (1.9–2.4)		
AUC_0–_*_t_* (h · mg/liter)	229.2 (30.9)	217.8 (18.4)		
AUC_0–∞_ (h · mg/liter)	230.6 (30.7)	221.2 (17.4)		
*t*_1/2_^*c*^ (h)	1.7 (0.9–2.8)	1.6 (0.9–1.8)		
*V*_ss_ (liters)	22.2 (42.0)	13.0 (17.8)		
CL (liter/h)	8.7 (45.5)	5.6 (16.0)		
CL/W (liter/kg/h)	0.169 (37.9)	0.226 (20.0)		
Avibactam				
*C*_max_ (mg/liter)	15.1 (52.4)	14.1 (23.0)	13.7^*b*^ (22.4)	16.3^*b*^ (22.6)
*t*_max_ (h)	2.0 (1.9–2.6)	2.1 (1.9–2.4)		
AUC_0–_*_t_* (h · mg/liter)	36.3 (33.7)	34.4 (23.4)		
AUC_0–∞_ (h · mg/liter)	36.4 (33.6)	34.8 (22.6)		
*t*_1/2_ (h)	1.6 (0.9–2.8)	1.7 (0.9–2.0)		
*V*_ss_ (liters)	31.0 (53.3)	19.3 (27.0)		
CL (liter/h)	13.7 (52.6)	8.9 (30.2)		
CL/W (liter/kg/h)	0.267 (44.2)	0.359 (35.8)		

a Values are geometric mean (coefficient of variation [%]) unless stated otherwise. CL/W, weighted clearance or clearance by body weight.

b Plasma concentration as measured at end of infusion.

c Median (range).

### Population pharmacokinetic modeling.

The final population PK models for ceftazidime and avibactam were two-compartment disposition models with first-order elimination from the central compartment, including the covariate effect of the allometric model for body weight and the age effect on renal maturation. Goodness-of-fit plots and visual predictive checks indicated that the population PK models accommodated the pediatric data well, with no obvious bias, and were considered sufficiently robust to support extrapolation to younger children for use in PTA simulations (42). AUC_0–∞_ values were estimated from population PK modeling; they were comparable across all 4 cohorts and in line with adult exposures (Table 4).

**TABLE 4:** Summary of ceftazidime and avibactam observed and population pharmacokinetic model-predicted exposures in pediatric patients (pharmacokinetic population)

Drug	AUC_0-∞_ (h · mg/liter)				
	Observed value for cohort^*a*^:		Predicted value for cohort^*b*^:		Value for adult reference population^*c*^ (*n* = 16)
	1 (≥12 to <18 yr)	2 (≥6 to <12 yr)	3 (≥2 to <6 yr)	4 (≥3 mo to <2 yr)	
Ceftazidime	230.6 (30.7)	221.2 (17.4)	255.32 (43.95)	286.27 (37.13)	289.0^*d*^ (15.4)
Avibactam	36.4 (33.6)	34.8 (22.6)	43.25 (12.14)	48.99 (10.64)	42.1^*e*^ (16.0)

a Data for cohorts 1 and 2 are geometric means (percent coefficients of variation).

b Data for cohorts 3 and 4 are means (SD) based on population pharmacokinetic model predictions (42).

c Values are geometric means (percent coefficients of variation) for observed exposures from a phase I study in healthy adult volunteers on day 1 after receiving a single dose of ceftazidime-avibactam (2,000 to 500 mg) (44).

d *n* = 15.

e *n* = 13.

### Safety.

A summary of AEs is shown in Table 5. Overall, 6 patients (18.8%) reported a total of 9 AEs with onset after the start of the infusion of ceftazidime-avibactam. No AEs were reported in cohort 1 or cohort 2. Four patients (50%) reported at least 1 AE in cohort 3; 2 patients (25%) reported at least 1 AE in cohort 4. All AEs were of mild or moderate intensity. The most common AEs reported were gastrointestinal, occurring in 3 (9.4%) patients. Only 1 AE was reported that was considered related to the study drug: sinus tachycardia in a 14-month-old male patient in cohort 4. The patient entered the study with medical conditions including cervical lymphadenitis, wound infection (staphylococcal), and fever but no history of cardiovascular conditions. The event was of mild intensity, began approximately 1 h after the start of the ceftazidime-avibactam infusion, and lasted approximately 0.5 days. After a full review of this case, no new safety concerns were identified. No deaths or serious AEs were reported in the study.

**TABLE 5:** Summary of AEs in cohorts 3 and 4 (safety analysis population)^*c*^

System organ class/preferred term	No. (%) of patients with AE for cohort^*b*^:		
	3 (≥2 to <6 yr)(*n* = 8)	4 (≥3 mo to <2 yr)(*n* = 8)	Total^*a*^ (*n* = 32)
Any	4 (50.0)	2 (25.0)	6 (18.8)
Cardiac disorders	0	1 (12.5)	1 (3.1)
Sinus tachycardia	0	1 (12.5)	1 (3.1)
Gastrointestinal disorders	3 (37.5)	0	3 (9.4)
Constipation	1 (12.5)	0	1 (3.1)
Diarrhea	1 (12.5)	0	1 (3.1)
Vomiting	1 (12.5)	0	1 (3.1)
Investigations	1 (12.5)	0	1 (3.1)
Increased alanine aminotransferase	1 (12.5)	0	1 (3.1)
Increased aspartate aminotransferase	1 (12.5)	0	1 (3.1)
Increased blood triglycerides	1 (12.5)	0	1 (3.1)
Increased gamma-glutamyltransferase	1 (12.5)	0	1 (3.1)
Injury, poisoning, and procedural complications	0	1 (12.5)	1 (3.1)
Procedural site reaction	0	1 (12.5)	1 (3.1)

a Total patient number includes cohorts 1 to 4.

b Onset on or after the date and time of the first dose of ceftazidime-avibactam and up to and including the day 3 follow-up visit.

c No AEs were reported in cohorts 1 and 2.

No potentially clinically significant hematology, clinical chemistry, or liver function tests were reported, and there were no clinically significant changes in electrocardiograms as determined by the study investigators.

## DISCUSSION

This phase I trial was the first study to evaluate the PK profile, safety, and tolerability of a single dose of ceftazidime-avibactam in hospitalized pediatric patients. The study allowed successful characterization of the single-dose PK profile of ceftazidime and avibactam in a pediatric population aged from 3 months to <18 years who were already receiving systemic antibiotic therapy for suspected or confirmed infection.

Ceftazidime and avibactam plasma PK parameters of *C*_max_, AUC_0–*t*_, AUC_0–∞_, *t*_max_, and *t*_1/2_ were similar between cohort 1 (≥12 to <18 years) and cohort 2 (≥6 to <12 years) for both drugs. Ceftazidime and avibactam plasma concentration profiles were similar in cohorts 1 and 2 across the sampling time points. Based on the sparse blood sampling and overlay of available plasma concentration data against time for the 4 cohorts, observed mean plasma concentrations appeared similar in cohorts 3 (≥2 to <6 years) and 4 (≥3 months to <2 years) to those observed in cohorts 1 and 2.

The PK profiles of single-dose ceftazidime and avibactam in this hospitalized pediatric population were similar to those previously observed in an adult population. In a phase I study in healthy adults (44), geometric mean *C*_max_ and total exposures (AUC_0–∞_) following a single 2-h i.v. infusion of ceftazidime-avibactam at 2,000 mg/500 mg were 88.1 mg/liter and 289 mg · h/liter, respectively, for ceftazidime and 15.2 mg/liter and 42.1 mg · h/liter, respectively, for avibactam. In the current study, peak exposure (*C*_max_) of both ceftazidime and avibactam in cohorts 1 and 2 was comparable to that previously described for the adult population; however, total exposures were somewhat lower than those for the adult population (approximately 40 mg · h/liter lower for ceftazidime and approximately 4.5 mg · h/liter lower for avibactam when comparing cohorts 1 and 2 to adults).

Due to the sparse sampling in cohorts 3 and 4, population PK modeling was used to estimate AUC_0–∞_ to enable comparison across all cohorts. The population modeling provided a good estimate of study drug exposure, with simulated study drug exposures (AUC) similar across all four age cohorts for avibactam and ceftazidime and broadly comparable with previously observed data in adults.

This study did not identify any new safety concerns, and the data supported the decision to initiate multiple-dose phase II treatment studies investigating the safety of ceftazidime-avibactam in hospitalized pediatric patients with cIAI (NCT02475733) or cUTI (NCT02497781). Further PK/PD modeling has been completed using the PK data generated in the present study and will be described separately. This compares modeled exposures in pediatric patients to those in adults and predicts PTA against the preclinical targets associated with efficacy in adults. Together, these data have been used to inform dose selection for the planned phase II studies of ceftazidime-avibactam in pediatric patients.

### Conclusions.

The data from this study were sufficient to characterize the PK profile of ceftazidime and avibactam in pediatric patients hospitalized with infection who received a single-dose i.v. infusion of ceftazidime-avibactam. PK profiles of both study drugs were comparable across all 4 age cohorts and were broadly similar to those previously observed in adults. No new safety concerns were identified. In addition, these data support the progression to phase II trials of ceftazidime-avibactam in pediatric patients and have been instrumental in the development of population PK modeling and subsequent PTA simulation that has supported dose decisions in these phase II studies.
